# Wrinkled Thermo-Electric Meander-Shaped Element on a Thin Freestanding PDMS Membrane

**DOI:** 10.3390/membranes13050508

**Published:** 2023-05-11

**Authors:** Liubov Bakhchova, Liudmila Deckert, Ulrike Steinmann

**Affiliations:** Institute for Automation Technology, Faculty of Electro Engineering and Information Technology, Otto-von-Guericke University Magdeburg, 39106 Magdeburg, Germany; liudmila.deckert@ovgu.de (L.D.); ulrike.steinmann@ovgu.de (U.S.)

**Keywords:** polydimethylsiloxane, metallization, ultra-thin freestanding membrane, meander, wrinkle

## Abstract

Natural wrinkling of metal films on silicone substrates can appear by means of the metal sputtering process and can be described by the continuous elastic theory and non-linear wrinkling model. Here, we report the fabrication technology and behavior of thin freestanding Polydimethylsiloxane (PDMS) membranes equipped with thermo-electric meander-shaped elements. The Cr/Au wires were obtained on the silicone substrate by magnetron sputtering. We observe wrinkle formation and suppose furrows appear once PDMS returns to its initial state after the thermo-mechanical expansion during sputtering. Although the substrate thickness is usually a negligible parameter in the theory of wrinkle formation, we found that the self-assembled wrinkling architecture of the PDMS/Cr/Au varies due to the membrane thickness of 20 µm and 40 µm PDMS. We also demonstrate that the wrinkling of the meander wire affects its length, and it causes a 2.7 times higher resistance compared to a calculated value. Therefore, we investigate the influence of the PDMS mixing ratio on the thermo-electric meander-shaped elements. For the stiffer PDMS with a mixing ratio of 10:4, the resistance due to wrinkle amplitude alterations is 25% higher compared to the PDMS of ratio 10:1. Additionally, we observe and describe a thermo-mechanically induced motion behavior of the meander wires on completely freestanding PDMS membrane under applied current. These results can improve the understanding of wrinkle formation, which influences thermo-electric characteristics and may promote the integration of this technology in applications.

## 1. Introduction

The recent development of lab-on-chip applications has highlighted the need to implement electrodes on flexible and stretchable materials, such as Polydimethylsiloxane (PDMS) [1,2,3]. This silicone is widely used for medical applications due to its unique properties. It is transparent, flexible, gas permeable, and easy to structure [4]. One of the promising research directions for PDMS-based applications is Organ-on-chip [5,6]. It is a microfluidic platform with various channels used for cell culture purposes. The main advantage of such a platform over the classical approach using a Petri dish is the possibility to emulate a dynamic microfluidic environment in a well-controlled, reproducible, and reconfigurable manner [7]. Thus, the human body’s relevant conditions can be mimicked. However, due to the miniature size of the compartments and the encapsulation of the system (access to the channels is possible only via tiny mm sized inlets and outlets at the chip edges), it is impossible to insert the necessary measurement equipment for a Petri dish or Boyden chamber, e.g., Electrical Resistance System. However, measurements of the physiological and biological parameters in the direct vicinity of the cells (in situ) are required to characterize tissue functionality and cells’ reactions, e.g., in the presence of drugs [1]. Thus, the next disruptive research direction in Micro-Electro-Mechanical Systems (MEMS) is sensor integration into a microfluidic platform for cell culture [8,9,10,11]. In addition, it is of great interest to integrate various actuator functionalities, which can help to manipulate liquid or cells [12].

Depending on which medical or biological question we address through the Organ-on-Chip application, we might need to vary in situ temperature, e.g., for inflammation modeling. Therefore, we need to heat parts of the structure locally and most efficiently. As mentioned previously, PDMS is the most common silicone used for the realization of Organ-on-Chip devices. It has several interesting properties related to thermal behavior, e.g., PDMS gas permeability is firmly temperature-dependent. This effect can be used in applications that can take advantage of varying permeability by heating the PDMS substrate. If a heating element, which is essentially an electrode with specific target resistance, is directly applied on the PDMS surface, one can heat PDMS locally with high efficiency.

It is possible to structure metal electrodes on flexible polymers, such as polycarbonate (PC) or polyester (PET), and several research groups have reported sensor fabrication on these materials. For example, Henry et al. [13] have reported gold electrodes patterned onto polycarbonate substrates for Transepithelial Electrical Resistance (TEER) measurements in a combined PC/PET/PDMS Organ-on-chip structure. In the case of a pure PDMS-based realization, the metal electrodes must be patterned on flexible and stretchable silicone. This poses additional significant research challenges on micro-structuring, such as micro-cracks, delamination, and potential degradation of electromechanical behavior caused by mechanical deterioration [14]. In addition, metal deposition by magnetron sputtering can cause wrinkles/grooves on the PDMS surface, which in turn are transferred to the adsorbed metal [15]. This effect may be due to the thermo-mechanical expansion of the PDMS during the metal deposition process and its relaxation [16]. The authors of [17] deposited Molybdenum on elastic PDMS, showing that naturally created wrinkles’ wavelength increases linearly with the metal film thickness. Their results are in good agreement with the continuous elastic theory. A similar surface modification, but more controlled, can be achieved by pre-stretching PDMS. This method can enable electrode “self-healing” in case of mechanical movements. Thus, the authors of [18] deposited Zn nano-film on the pre-stretched PDMS using a direct current magnetron sputtering.

Interestingly, the authors of [19] describe wrinkles which appear only close to cracks of the metal film. They applied DC magnetron sputtering of iron on uncured and partially cured PDMS substrates on a glass bottom layer. The authors of [20] created a tunable metal film thickness gradient to better understand the ordered wrinkling patterns. They observed coexisting branched stripes, herringbones, and labyrinths along the direction of thickness decrease and explained it using a non-linear wrinkling model. The authors also fabricated tailored wrinkles based on Cu and thin PDMS [21]. Promising applications for tailored wrinkled soft metalized layers [22,23] include strain sensors [24] and droplet motion devices [25]. However, considering the published works, there is no clear and univocal understanding of the wrinkle formation mechanism and behavior of large-area freestanding metallized soft polymer layers. In addition, no studies have been conducted on the dependence of wrinkle creation on the stiffness of Polydimethylsiloxane.

Therefore, in this work, we report the fabrication methodology and thermo-electrical and thermo-mechanical behavior of the first-time realized Cr/Au thermo-electric structure on a thin freestanding Polydimethylsiloxane membrane. In addition, we developed meander-shaped elements with different wire lengths to achieve higher resistance values. In this work, we also report natural wrinkle formation during magnetron sputtering on soft layers with various thicknesses of 20 µm and 40 µm. Moreover, we present the temperature–current and resistance–current characteristics of the meanders on freestanding and fixated PDMS layers. To the best of our knowledge, we investigate and report for the first time the influence of the substrate hardness on the thermo-electric characteristics of the metal structures on it. Therefore, we explore the impact of the 10:1 and 10:4 mixing ratios of the PDMS’s base and curing agent on the meanders’ thermo-electrical characteristics. Additionally, this paper investigates the PDMS/Cr/Au interface regarding naturally created wrinkles and discusses the physical background of its formation. Last but not least, we report the mechanical deformation of the PDMS freestanding membrane and the meanders’ movement by applying DC current. 

## 2. Materials and Methods

### 2.1. Fabrication and Metallization of the Thin PDMS Membrane

This work is based on the following samples, which differ in PDMS thickness of 20 µm and 40 µm; PDMS mixing ratios of 10:1 (soft) and 10:4 (hard); and meanders’ lengths of 29 mm (short) and 48 mm (long). 

**Figure 1:**
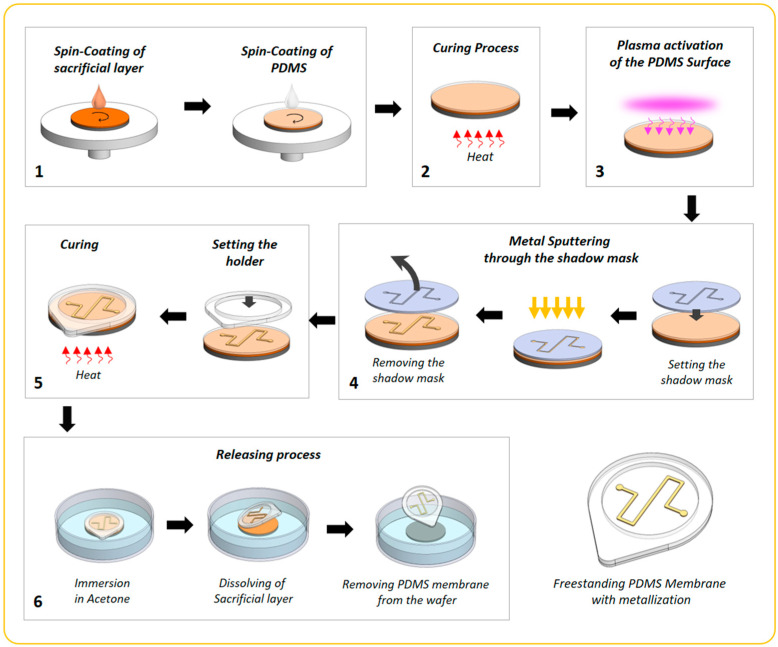
Schematic description of the fabrication process for a thermo-electric meander-shaped element on a thin freestanding PDMS membrane. Technological steps: (1) spin coating of photoresist as a sacrificial layer with a following spin coating of PDMS layer; (2) curing process in the oven; (3) oxygen plasma treatment of the cured layers; (4) metal sputtering through a shadow mask; (5) attaching the ring holder and curing it in the oven at 60 °C for 3 h; and (6) releasing membrane by rinsing in acetone, which dissolves the sacrificial layer.

A detailed schematic description of the developed technology is given in Figure 1. A 1′ silicon wafer was utilized as a substrate for thin PDMS membranes. The technological process for obtaining thin freestanding PDMS membranes is a sequence of the following steps. First, all wafers were cleaned by rinsing them in isopropanol and dehydrated at 200 °C for 5 min. Next, the photoresist AZ 1512 (Microchemicals GmbH, Ulm, Germany) was used as a sacrificial layer from which the membrane can be released by immersing it in acetone. The sacrificial layer was obtained using a spin coater POLOS Spin150i (SPS-Europe B.V., Putten, The Netherlands) at 2000 rpm for 20 s to realize a 2.5 µm thick coating, following soft baking on a hot plate at 100 °C for 1 min. These silicon substrates with sacrificial layers were utilized to fabricate all the following samples.

To obtain the PDMS membrane, a solution of PDMS base-polymer (Sylgard 184, Dow Corporate, Midland, MI, USA) with a curing agent (10:1 and 10:4 *w*/*w* mixing ratio) was prepared and spin-coated on the surface of the sacrificial layer. A higher concentration of the curing agent results in a harder outcome material. Spin-coating parameters (rotation speed and time) were varied to obtain PDMS membranes of 20 µm and 40 µm thickness. These data are shown in Figure S1 (Supplementary Document). The thickness of the PDMS layer was measured using a spectrophotometer F20e-UVX (Filmmetrix Inc., San Diego, CA, USA). Then, all samples were placed in the oven at 60 °C for two hours. Once the PDMS was cured, the layers were treated with oxygen plasma (ATTO Plasma Cleaner, Diener electronic GmbH & Co. KG, Ebhausen, Germany) for 20 s at 70% power of the generator and 0.5 bar gas pressure in the chamber.

Metallization on the PDMS membrane surface was carried out by using magnetron sputtering of Cr and Au through a shadow mask, while the PDMS layer was located on the silicon wafer. A shadow mask with a meander-shaped opening was laser cut from the 300 µm thick magnetic steel, placed on the sample, and held by the magnet at the bottom side of the silicon wafer. The metal deposition process lasted 30 s of sputtering and 5 min of cooling time. Thus, 14 steps were performed: 2 for chromium at 30 W (100 mA) and 12 for gold at 60 W (100 mA). Argon was utilized as process gas, and the pressure in the chamber reached 3.5 × 10^−3^ bar. Cr was utilized as an adhesive layer.

A PDMS ring holder was fabricated and used to better handle and release the thin membranes. Therefore, a 1 mm thick PDMS layer was cured on a 4′ silicon wafer, released, and cut into a circle with an inner diameter (ID) of 2 cm and outer diameter (OD) of 2.7 cm. Subsequently, a small amount of liquid PDMS was distributed on the ring holders. They were placed on cured membranes and baked in the oven for 1 h at 60 °C. Finally, the sacrificial photoresist AZ 1512 layer was removed by immersing it in acetone and rinsing it for 1 min. Therefore, the PDMS membrane with a meander-shaped metal structure is removed from the wafer and becomes freestanding. Nevertheless, the ring holder serves only as a holder for an ultra-thin PDMS membrane and can be cut off if the layer is applied to any system, e.g., one of the lab-on-chip structures.

### 2.2. Measurements and Analysis

The resistance of the meander wires on PDMS membranes was characterized while layers were on the silicon wafer and after release. Both measurements, non-freestanding and freestanding, were carried out on a prepared Teflon block with a cavity for samples and golden pin contacts built into it (Figure S2). The sample is placed on top of spring pin contacts. Their exact position and gravitation ensure soft mechanical contact with the meanders’ contact pads. The current was applied in the range from 20 mA to 180 mA with step 20 mA, and voltage was recorded. Therefore, the resistance values were determined. Theoretical resistance values of the modeled and CAD plotted (AutoCAD Mechanical 2020, Autodesk Inc., San Francisco, CA, USA) meanders were calculated according to Pouillet’s law, and the planar wire without wrinkles was considered. During the current application, temperature measurements were carried out using a portable infrared camera (HT-A1, Dongguan Xintai Instrument Co., Guangdong, China). Temperature measurements were taken 10 s after the current was turned on to ensure a balance between the thermal energy supplied and the thermal energy released to the environment.

The surface analysis and wire deformation characterization were performed using optical microscopy (VHX—500F, Keyence Deutschland GmbH, Neu-Isenburg, Germany). Surface roughness and metal thickness were characterized using a 3D profilometer (VR-6000, Keyence Deutschland GmbH, Neu-Isenburg, Germany). The adhesion force was characterized using the pull-off test. Metal on PDMS was glued to the dolly using epoxy adhesive “Uhu Plus Endfest 300”, and the breaking strength was measured using an XYZTEC Condor Sigma bond tester.

Hardness measurements were realized by means of a digital hardness tester HT-6510C (Precision Scientific Instruments Corp., Delhi, India). For this purpose, 30 × 30 mm^2^ and 6 mm thick PDMS blocks were prepared. The PDMS base and curing agent mixing ratios were 10:1 and 10:4, respectively.

## 3. Results and Discussion

According to the processes described above (Section 2.1), we have developed thermo-electric meander-shaped elements on a thin freestanding PDMS membrane. The samples described below were designed with the following intentions: PDMS membrane thickness was chosen to correspond to the one typically used in Organ-on-chip applications, which is in the range of 10 to 50 µm [3,5]; the geometric parameters of the thermo-electric element (wire width, length, and thickness) were set regarding the minimization of power consumption and ensuring power efficiency; and the mixing ratio of the PDMS was varied to improve the stiffness and handling of the thin membranes. 

First, we characterized the surface of the initially cured PDMS layers to analyze and describe the developed structures. Figure S3a shows it is smooth and replicates the substrate profile below. In our study, it is the silicon wafer with the spin-coated photoresist. As it occurs from our experience and in correlation with other works [23], oxygen plasma treatment of PDMS makes its surface hydrophilic and improves the adhesion to metal. Therefore, Figure S3b shows the PDMS surface after 20 s of oxygen plasma treatment. Even if wrinkles or deformations because of thermal expansion appeared during the plasma process at the blank PDMS layer, they must have vanished directly after it. Therefore, the surface shown is homogenous and planar. Moreover, the overall PDMS thickness variations were less than 2% of the respective membrane thickness. The precise values are given in Table S1. In addition, the PDMS thickness inhomogeneity in the frame of each sample stays in the sub-micron level and is ±0.16 µm (Table S2).

**Figure 2:**
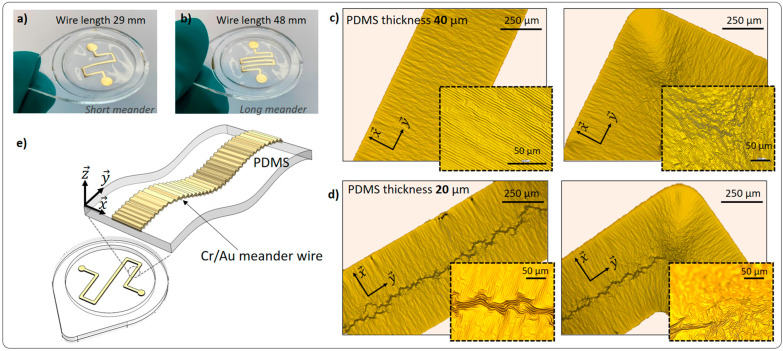
Photographs of freestanding PDMS membranes with (**a**) short meander (29 mm) and (**b**) long meander (48 mm); (**c**,**d**) are the optical images of the meander wires deposited on 40 and 20 µm thick PDMS layers, respectively; (**e**) is the 3D model of the thin membrane with the thermo-electric element, where the wrinkling of the PDMS/Cr/Au structure is qualitatively shown in magnification. The sample’s dimensions are given in associated Figure S4 in the Supplementary File.

As shown in Figure 1, the next technological step is metal deposition. Hereof, we have sputtered the meander-shaped structures with 29 mm and 48 mm wire lengths through a shadow mask onto thin PDMS layers, shown in Figure 2a,b, respectively. The resulting thickness of Cr, which is used as an adhesive layer, was 25 nm, and the resulting thickness of gold was 409 nm. The pull-off test shows a high adhesion rate of Cr/Au thin film to oxygen plasma-treated PDMS. The breaking strength value is 1.4 MPa.

After the magnetron sputtering, which is the argon plasma process, we observe wrinkled metal wires with one-directional ($\vec{x}$) and two-directional deformations ($\vec{x}\;and\;\vec{y}$), as it is shown in Figure 2c,d. The one-dimensional wrinkles were always induced parallel to the $\vec{x}$ direction with a wavelength λ of approximately 5 µm and amplitude *A* of 0.27 µm (Figure S5). In the corner of the meander, two directions are parallel to the wire width and converge. Therefore, two-directional wrinkles are formed. If a metal layer is deposited on PDMS, the polymer surface expands while being slightly heated. It should be noted that the overall temperature in the vacuum chamber does not exceed 30 °C, since we performed interval sputtering (Section 2.1). We assume that the reason lies in the ejection of target metal atoms, which have sufficient kinetic energy to reach the substrate and disperse the rest of their energy, which is then converted into heat. Due to the heating that takes place on the surface of PDMS, a thermally induced mechanical expansion takes place. We observe this for the samples of 20 µm and 40 µm thickness. After cooling, the surface of PDMS—now with deposited metal on it—returns to its initial state, and wrinkles are formed. The result is coherent with the state of the art [20,21,22,23,24,25]. Therefore, the PDMS/metal wrinkles, shown in Figure 2c,d, result from the plasma treatment which occurs in the course of sputtering. In our study, the wrinkles on metallized PDMS are formed naturally, without any pre-stretching of the PDMS.

Furthermore, in Figure 2c,d, we compare the wrinkles on the wires sputtered onto 40 µm and 20 µm thick PDMS, respectively. In both cases, the thickness of the metal is identical. As it is seen in Figure 2d, in the middle of the wire on 20 µm thick PDMS, a $\vec{y}$ prolonged wrinkle is present. However, this wrinkle cluster is absent on the 40 µm thick PDMS membranes. According to a theory in [26], hierarchical wrinkles are generated due to the metal thickness gradient, which can appear at the metal deposition through the shadow mask. In our case, two metal thickness gradients are at opposite sides of the meander wire. Therefore, hierarchical wrinkles are created simultaneously from two opposite directions and meet in the middle of the wire. Then, the perpendicular grooves are formed due to the differences in wavelength, amplitude, and period of the two wrinkle clusters. However, this effect is not seen on the 40 µm PDMS membrane (Figure 2c) and thicker membranes, e.g., 1 mm (Figure S6). Obviously, the difference is due to the thickness of the soft substrate, but this parameter is not present in the wrinkling theory. In both the continuum elasticity theory [20] and the non-linear wrinkling model [26], the system is usually treated as a thin, rigid film resting on a semi-infinitely thick, compliant substrate under elastic deformation. Furthermore, the ($\vec{x}\;and\;\vec{y}$) labyrinth-like wrinkles are observed on the contact pad areas of the metal wire (Figure S7), which have circular geometry with a diameter of 3 mm. The radial compression and isotropic thermal expansion of the PDMS resulted in hierarchical wrinkling over the entire circular area. Interestingly, the wrinkle pattern on the edge of the circle (Figure S7a,b) is similar to the radial hierarchical wrinkle domains, obtained by the solvent evaporation method, presented in [27]. At the same time, the middle part of the pad is filled with the labyrinth-like wrinkles, which are dependent on the elasticity of the substrate and deposited metal thickness, presented in several works [17,21,27,28]. Comparable wrinkling behavior was also observed on the sputtered without shadow mask circular thick samples (Figure S8). The circular PDMS substrates of thickness from 0.1 mm to 1 mm with 0.1 mm step were entirely metalized and radial hierarchical wrinkling patterns were observed.

**Figure 3:**
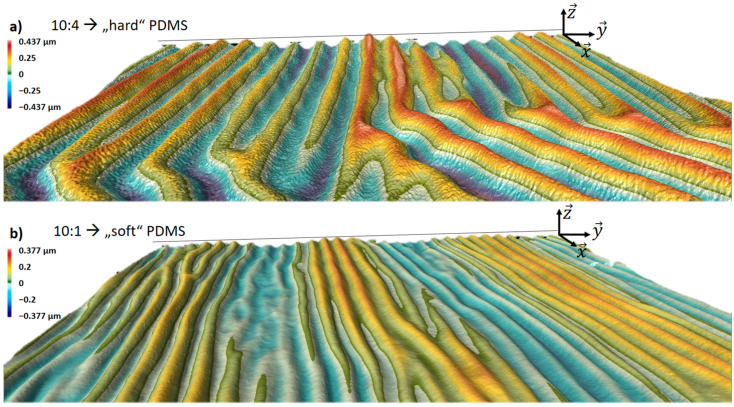
Profile characterization of the Cr/Au meanders on the 40 µm thick freestanding PDMS membranes with different mixing ratios of 10:4 (**a**) and 10:1 (**b**) PDMS base and curing agent, respectively.

In the previous paragraph, we discovered the influence of the substrate geometry, but we also studied the influence of the PDMS material properties on metal wrinkling architecture. Accordingly, by manipulation of PDMS stiffness, we expected to increase its mechanical stability. Therefore, we fabricated identical samples to the previously described ones, but with the mixing ratio of 10:4 PDMS base and curing agent. It resulted in a PDMS hardness of 74.6 ± 0.1 HC, which is higher than that of samples with a mixing ratio of 10:1, with a hardness of 70.4 ± 0.1 HC. The surface, as expected, was partially flat without wrinkles (Figure S9), but the wrinkled and non-wrinkled islands were irregularly alternated. Nevertheless, the wrinkled area of the PDMS/metal was dominating. Surprisingly, the wrinkles’ amplitude resulted in a higher value and was 0.437 µm for the 10:4 PDMS. Comparably, the average amplitude of the wrinkles on 10:1 PDMS was 0.26 µm. The roughness comparison of both PDMS types is shown in Figure 3 and Figure S7. 

As described, wrinkles are generated by means of the PDMS thermo-mechanical expansion. Therefore, the entire meander wire is wrinkled (Figure 2e), and its length is extended compared to the design parameters. Thus, a difference in calculated and measured meander resistance appears. The DC resistance of the shorter thermo-electric element, described in Figure 3b, was measured to be approximately 11 Ω, while its calculated value is 3.5 Ω (factor of 3.14). Likewise, the calculated resistance of the longer device in Figure 2b is 5.73 Ω, and its measured value is 14 Ω, which is 2.44 times higher due to the wrinkled structure of the wire. We estimate this tendency under the condition of unchanged wire thickness and width, as well as the resistivity of the metal. 

**Figure 4:**
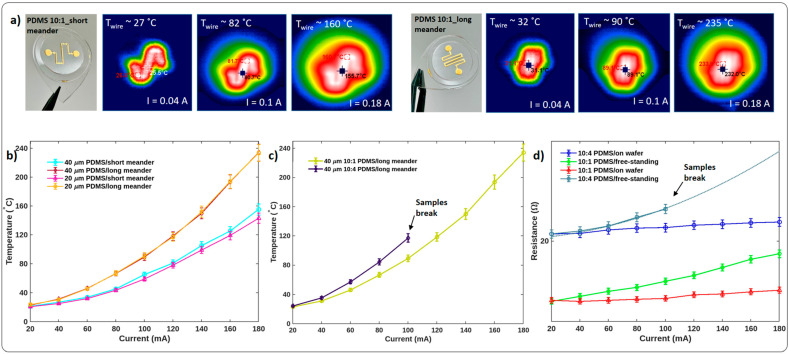
Photographs from an IR camera (**a**) of the short (**left**) and long (**right**) meander on a 20 µm thin freestanding PDMS membrane at different applied currents, which are 0.04 A, 0.1 A, and 0.18 A from left to right, respectively. (**b**) Temperature/current characteristics of long/short meanders (each 10:1 mixing ratio). (**c**) Temperature/current characteristics of long meanders on 10:1 and 10:4 PDMS. (**d**) Resistance/current characteristics of the long meander-shaped structures on the 20 µm thin PDMS.

Figure 4a,b shows temperature measurements of the Cr/Au thermo-electric elements on 20 µm thin freestanding 10:1 PDMS membranes. At 180 mA, the wire temperature was approximately 160 °C for a short meander and 230 °C for the longer version. The temperature increases in a parabolic fashion, while the current growth corresponds to Joule’s first law. Consequently, due to the different resistances of short and long meanders, the slope of both classes of curves is also different. As we see in Figure 4b, PDMS thickness does not affect the temperature behavior. However, the stiffness of the PDMS has an influence, as it is seen in Figure 4c.

As mentioned previously and shown in Figure 3, the wrinkle height of the 10:4 PDMS differs significantly from that of the 10:1 PDMS, by a factor of 1.68. Accordingly, the resistance of a meander of equal length sputtered onto hard PDMS is higher than that on soft PDMS. Therefore, the achieved temperature at the same applied current is significantly higher, as shown in Figure 4c. The developed surface has a bigger area due to the wrinkles, as opposed to the purely planar case without wrinkles resulting in higher resistance. The resistance of the meander-shaped conductors realized on 10:4 PDMS is higher (20.7 Ω) than that of those realized on 10:1 PDMS (14.1 Ω). The resistance differs by a factor of 1.46. Thus, as shown in Figure 4c, the temperature is also higher, so the change is proportional.

Furthermore, the resistance was measured while samples were located on the silicon wafer before releasing and in the freestanding condition after releasing. Figure 4d shows that the presence of the silicon wafer does not influence the basic resistance, and curves start from the same resistance values. While applying current, we observe a different mechanical behavior of both membranes, since a freestanding membrane shows strong mechanical deflection, whereas a fixed membrane remains non-deformed. Thus, we must conclude that a thermally induced expansion/deflection takes place. The meanders’ and then the PDMSs’ temperature increases and resistance grows. The resistance of the freestanding 10:1 PDMS samples changes non-linearly and increases from 14.1 Ω to 18.7 Ω with applied current up to 180 mA (green line in Figure 4d). The same but non-freestanding sample has less freedom to expand, and the heat is distributed into the silicon wafer underneath. Therefore, the resistance changes in a more linear manner and reaches 15.3 Ω at 180 mA (red line in Figure 4d).

The same phenomenon is seen for the samples on hard 10:4 PDMS, with an initial basic resistance of 20.7 Ω. Hence, the thermo-electric elements on freestanding membranes reach 23.2 Ω at 100 mA, while the resistance of the sample on the wafer is 21.3 Ω at 100 mA (and 22.3 Ω at 180 mA). Nevertheless, freestanding samples with a 10:4 mixing ratio could not be measured at higher currents and temperatures because they tend to break. These samples had an inhomogeneous structure with alternating wrinkled and planar areas (Figure S5). Therefore, microscopic cracks appear in the metal layer during the thermomechanical expansion of the PDMS, weakening the advantages of the wrinkled area. From this, a kind of self-healing effect of the metal layer in the area of the wrinkled substrate can be deduced, which is not so sensitive to thermally induced mechanical stresses. Interestingly, the resistance of the meanders on 10:1 PDMS and the wrinkles’ wavelengths, even after 60 on/off cycles, are reproducible. The resistance and wavelength values are given in Table S3. According to the state of the art, the wrinkles provide a self-healing effect of the metal structure on the elastic substrate. However, this effect is not present in the samples with a 10:4 PDMS mixing ratio, and meander-shaped elements break (Figure 4c) after a couple of loadings due to the generated cracks on the non-wrinkled islands (Figure S9).

**Figure 5:**
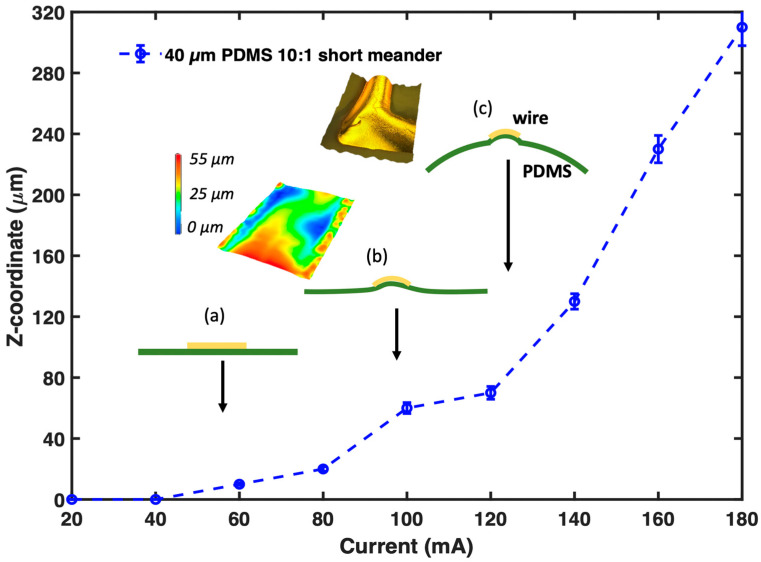
Graph representing the z-axis movement of the meander on the freestanding 40 µm thick membrane (**a**). Conceptual visualization inserts show the wire bending (**b**) and membrane deformation with wire bending (**c**).

Moreover, in the course of the thermo-mechanical expansion, we observe an actuator behavior of the entire PDMS membrane equipped with a thermo-electric element. The topographic survey during heating is shown exemplarily for a freestanding 40 µm thick 10:1 PDMS membrane in Figure 5. First, for the impressed DC current values ranging from 40 mA to 120 mA, we observe a mechanical movement of the wire (Figure 5, insets (a) and (b)). It bulges starting from a planar structure in the region of the metalized surface. We attribute this effect to the temperature differences between the wire and the PDMS environment. The area with the metal on top starts to expand due to the fast heating. Thus, the wire bends in a spheroidal manner. The middle part of the wire slowly goes up. At the same time, PDMS without metal does not deform and holds the edges of the wire at the start position. Thus, the height difference reaches 70 µm (compare *z*-coordinate of the diagram in Figure 5). Then, with a further increase in current from 120 mA to 180 mA, we observe the bulging of the entire membrane while the wire remains bent (Figure 5, inset (c)). The Supplementary Video S1 also shows the described membrane’s overall motion. Notably, the wrinkles do not smooth while the wire bends and membrane bulges.

It should be mentioned that the samples on a 20 µm thin freestanding PDMS membrane show different motion behavior. The previously described $\vec{y}$ prolonged wrinkle in the middle of the wire on 20 µm PDMS clearly influences the metal deformation: it buckles in the shape of a triangle, while the entire membrane continues to expand in the same curved shape as observed on the 40 µm PDMS membrane. 

## 4. Conclusions

In summary, thermo-electric meander-shaped elements of 29 mm and 48 mm on freestanding 20 µm and 40 µm PDMS membranes were fabricated and analyzed. PDMS is a soft silicone-based polymer. Therefore, natural wrinkles appeared on the material during magnetron sputtering of Cr and Au. The metal preserves the grooves, ensures the metal structure’s conductivity, and provides a self-healing effect even if the substrate deforms. Therefore, the samples are stable, and the measurements are reproducible even after 60 cycles of heating and passive cooling.

Furthermore, we showed the influence of substrate thickness on wrinkle formation. Therefore, the 20 µm and 40 µm PDMS membranes with metal meanders were compared. On a 40 µm thick layer, the wrinkles were one-directional ($\vec{x}$) except the meander corners, while on a 20 µm substrate, two-directional deformations ($\vec{x}\;and\;\vec{y}$) on the entire wire were present. According to the existing models, there are two clusters of hierarchical folds, with a $\vec{y}$ directed deformation forming in the center of the wire due to the collision of the metal gradient on both sides of the wire and the wrinkles’ phase shift. Since the wrinkle patterns differentiate due to the PDMS thickness, it is necessary to consider this parameter in the future development of wrinkle formation theories.

In addition, due to the wrinkled substrate, the length of the wire is increased. Therefore, the measured blank resistance is 11 Ω, compared to its calculated value of 3.5 Ω for a short version of the meander of 29 mm. Additionally, the measured resistance of a 48 mm long wire is 14 Ω instead of the calculated resistance of 5.73 Ω.

Moreover, we investigated the PDMS mixing ratio’s influence on the thermo-electric meander-shaped elements. The amplitude of the wrinkles on the more rigid PDMS with a 10:4 mixing ratio is higher (0.437 µm) compared to the 10:1 PDMS membrane with an amplitude of 0.27 µm. As we described in our work, the wrinkles extend the effective wire length; therefore, the resistance and temperature of the wire on 10:4 PDMS are higher than on 10:1 PDMS at the same applied current. At the same time, wrinkles ensure the conductivity and stability of the wire. The samples on hard PDMS have inhomogeneity and planar areas, where µ-sized cracks appear at a current of 100 mA. These breaks and delamination are absent on 10:1 PDMS samples. Hence, we showed the “self-healing” effect of the wrinkled metal layer.

Additionally, we observed and described the thermo-mechanical movement behavior of the meander wires and complete PDMS membrane with a thermo-electric meander-shaped element, which deforms in a domed shape at an applied current of 120 mA. In its turn, the wire bending depends on the wrinkle shape and positioning. Subsequently, further investigation is needed to ensure the integration of the meander-shaped thermo-electric element on freestanding thin PDMS membranes as a sensor or actuator in lab-on-chip applications, since the resistance depends on the wrinkles formed during the manufacturing process and the thermo-mechanical movement during the operating process.

## Data Availability

Not applicable.

## Supplementary Materials

The following supporting information can be downloaded at https://www.mdpi.com/article/10.3390/membranes13050508/s1, Figure S1: PDMS membrane thickness vs. rotation speed at different rotation times (2 and 4 min) for the solution of PDMS pre-polymer with a curing agent (10:1 *w*/*w* mixing ratio). Silicone is spin-coated on the silicon wafer with a prepared 2.5 µm photoresist sacrificial layer; Figure S2: Photographs of the Teflon test board for resistance and temperature measurements (a) without a sample on it and (b) with a sample on it; Figure S3: Optical microscope images of the PDMS surface before (a) and after (b) oxygen plasma treatment; Figure S4: Schematic representation of the obtained samples’ dimensions. In (a), the short (29 mm) meanders are shown, and in (b), the long (48 mm) meanders are shown (2D sketch and 3D visualization on 20 µm and 40 µm thick PDMS membrane); Figure S5: The graph representing the profile measurements by Profilometer Keyence VK-X3000; Figure S6: Cr/Au meander-shaped element on the 1 mm thick PDMS layer (a) with one-directional wrinkle pattern of the wire (b); Figure S7: Wrinkled contact pad surface of the developed meander-shaped element on 40 µm PDMS membrane for the mixing ratio (a,c) 10:1 and (b,d) 10:4. (c) and (d) present the magnified area in the middle of the contact pads shown in (a) and (b); Figure S8: Photograph of the sputtered Cr/Au on 10:1 PDMS test layers with a thickness range from 1 mm to 0.1 mm. The magnified surface view of the 0.1 mm thick PDMS with metal is on the right side. The wrinkle behavior is identical on all samples sputtered without a shadow mask; Figure S9: Optical microscope image of the Cr/Au meander surface. The substrate is 20 µm thick freestanding 10:4 PDMS membrane. Hard PDMS clearly shows planar and wrinkled areas. The µ-size cracks “self-heal” on the wrinkled region. Table S1: Detailed parameters of the thermo-electric meander-shaped elements on a thin freestanding PDMS membrane; Table S2: PDMS thickness distribution within the membrane area, where P1 is a measurement in the center, P3 near the edge, and P2 in between P1 and P3; Table S3: Resistance and wavelengths of the wrinkles for the 40 µm thick PDMS membrane with 29 mm meander after multiple on/off cycles. The intermediate time is 5 min. Video S1: Motion of the Wrinkled Thermo-Electric Meander-Shaped Element on a Thin Freestanding PDMS Membrane while applying an electric current (from 0 A to 0.18 A).
