# Supplementary material for: Wrinkled Thermo-Electric Meander-Shaped Element on a Thin Freestanding PDMS Membrane
Source: Membranes (Basel). 2023 May 11;13(5):508. doi: 10.3390/membranes13050508 (PMC10220571; doi:10.3390/membranes13050508)
Supplement: Supplementary file 1 [file membranes-13-00508-s001.zip › membranes-2362528-supplementary.pdf]

Supplementary Information

Wrinkled Thermo-Electric Meander-Shaped Element on a Thin Freestanding PDMS Membrane

Liubov Bakhchova *,†, Liudmila Deckert † and Ulrike Steinmann

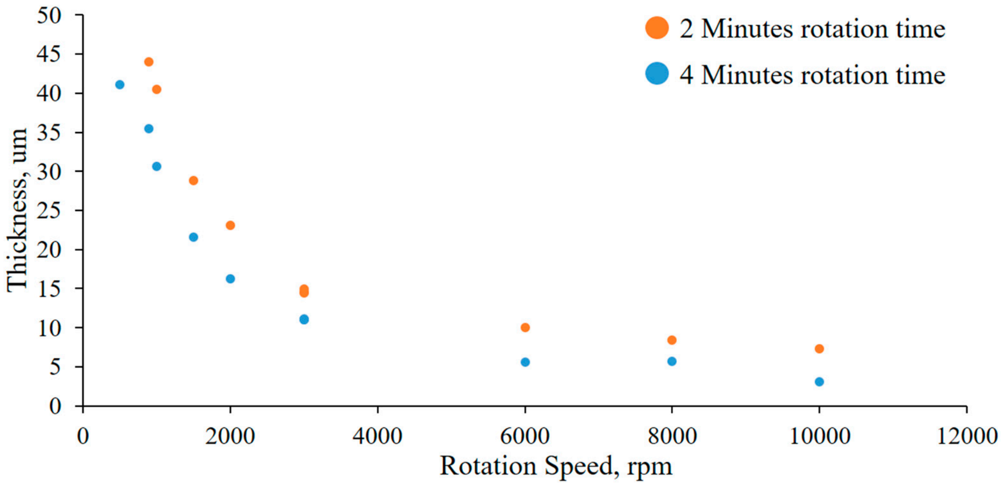

Figure S1. PDMS membrane thickness vs. rotation speed at different rotation time (2 and 4 minutes) for solution of PDMS pre-polymer with a curing agent (10 : 1 w/w mixing ratio). Silicone is spin-coated on the silicon wafer with prepared 2,5 μm photoresist sacrificial layer.

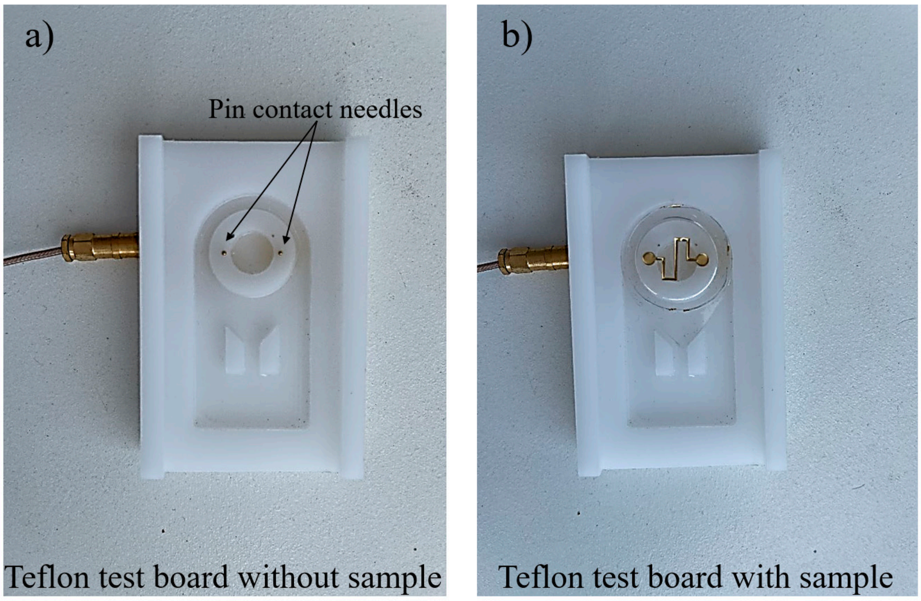

Figure S2. Photographs of the Teflon test board for resistance and temperature measurements (a) without sample on it and (b) – with.

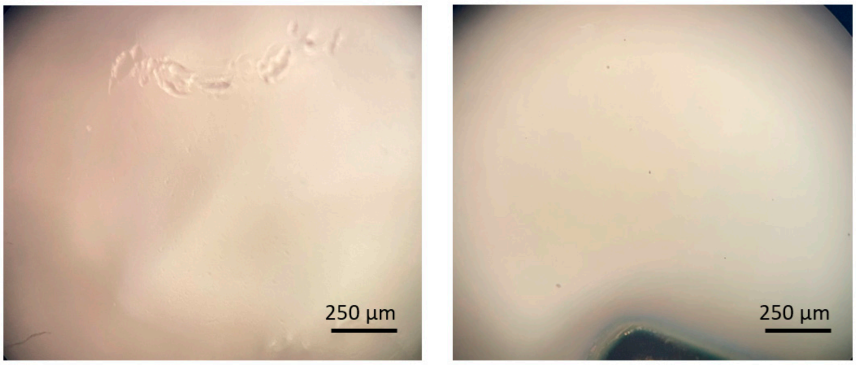

(a) **(b)**

Figure S3. – Optical microscope images of the PDMS surface before oxygen plasma treatment (a) and after (b).

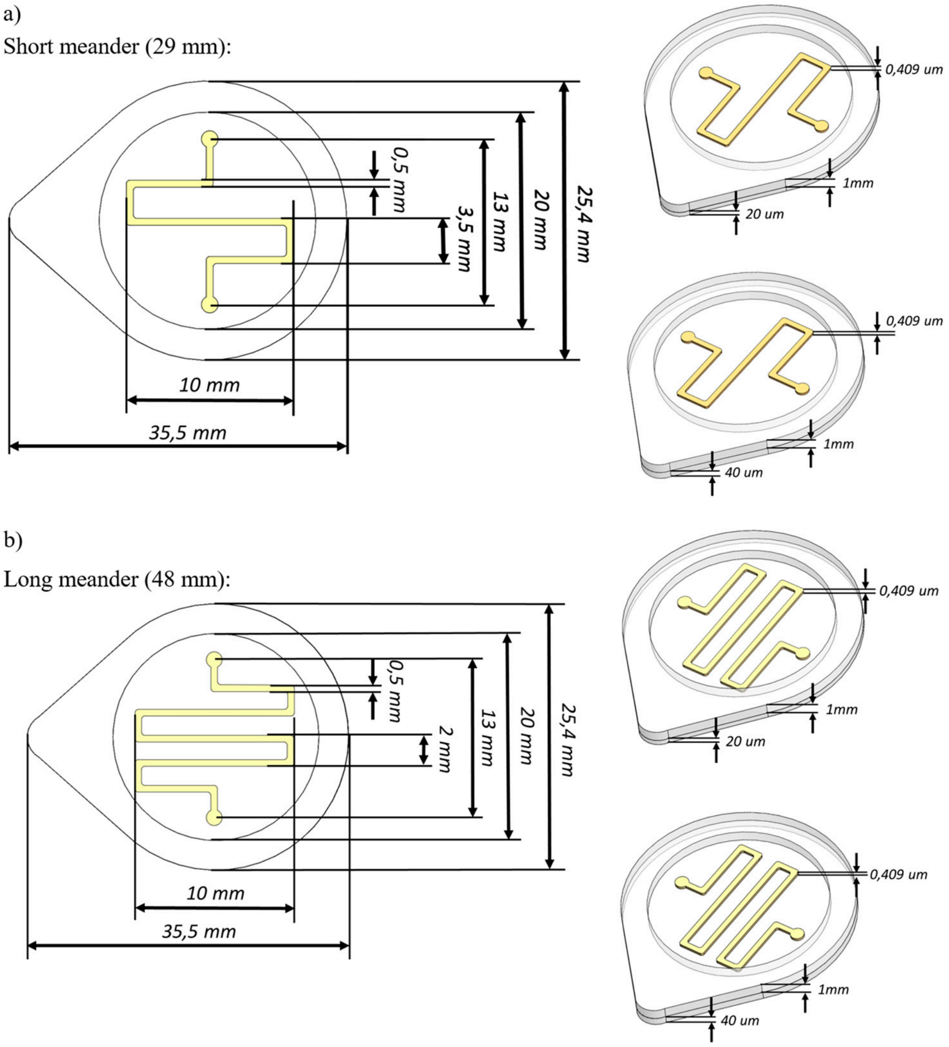

Figure S4. – Schematic representation of the obtained samples dimensions. In (a) the short (29 mm) and (b) the long (48 mm) meanders are shown (2D sketch and 3D visualization on 20 μm and 40 μm thick PDMS membrane).

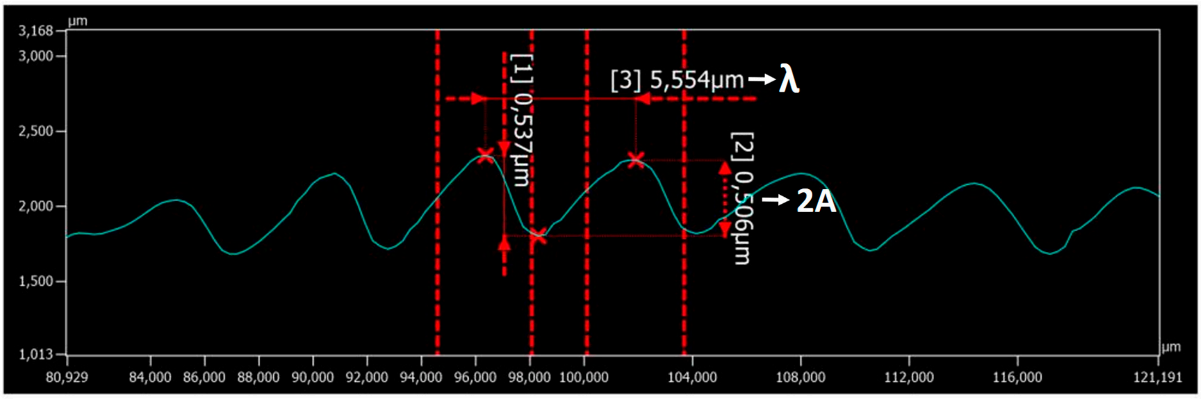

Figure S5. – The graph representing the profile measurements by Profilometer Keyence VK-X3000.

a)

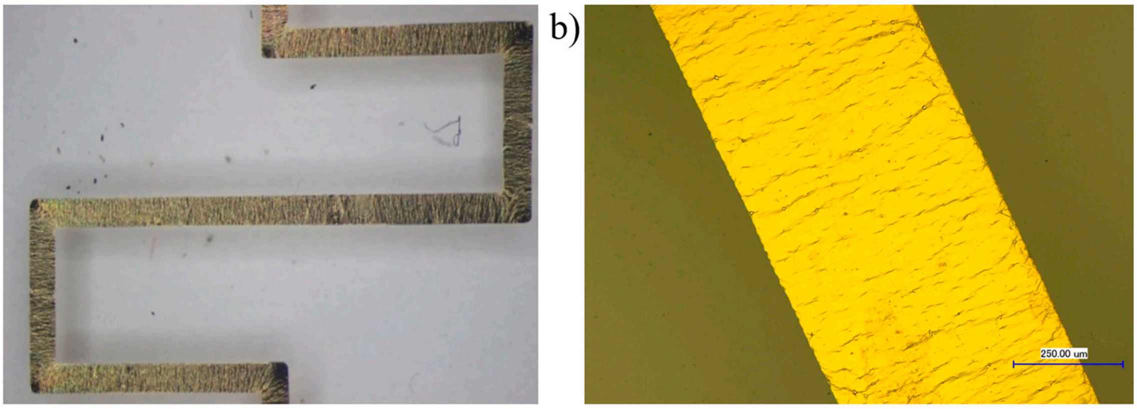

Figure S6. – Cr/Au meander shaped element on the 1 mm thick PDMS layer (a), with one-directional wrinkle pattern of the wire (b).

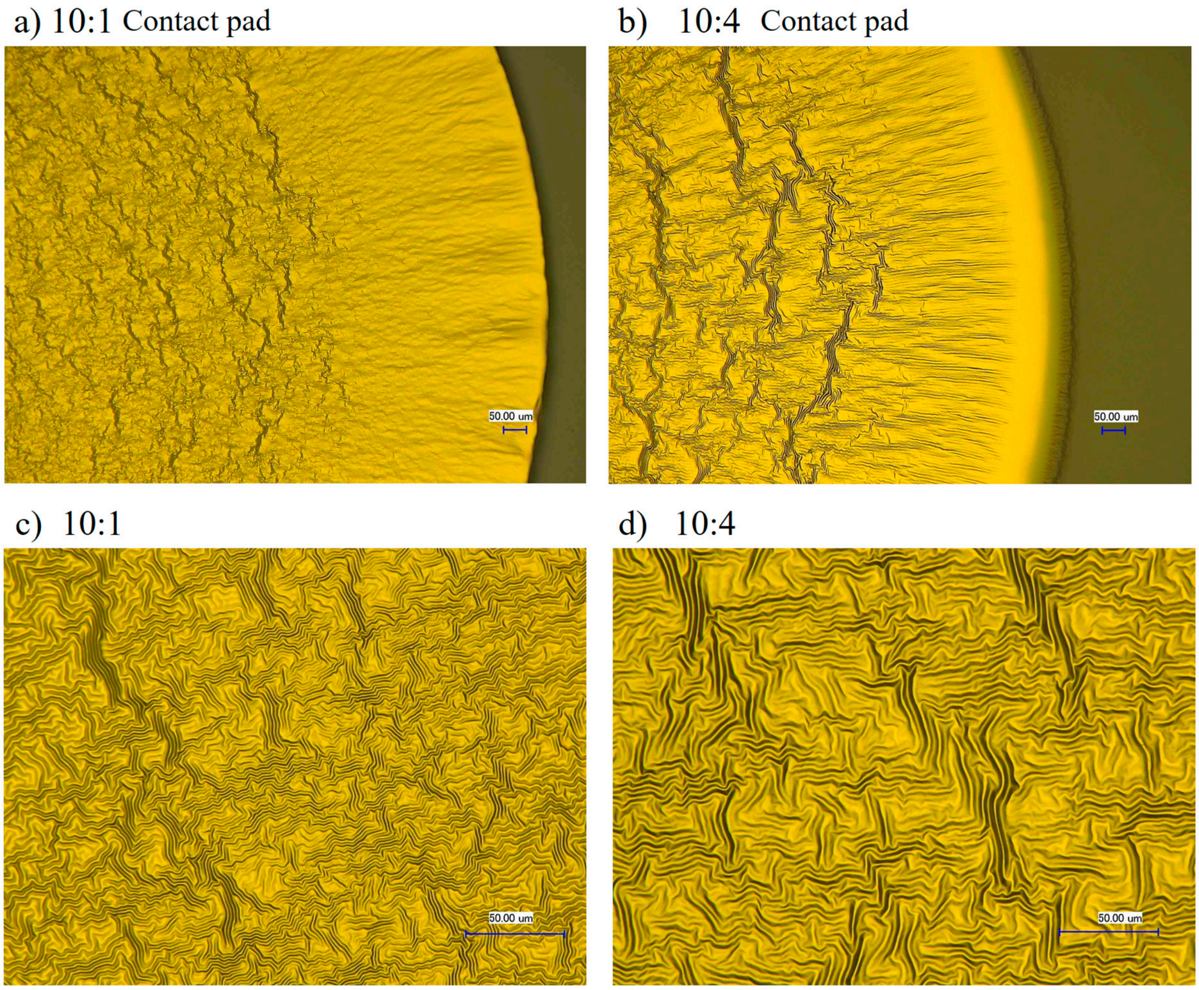

Figure S7. – Wrinkled contact pad surface of the developed meander-shaped element on 40 µm PDMS membrane, for the mixing ratio a, c) 10:1 and b, d) 10:4. The c) and d) present the magnified area in the middle of the contact pads shown in a) and b).

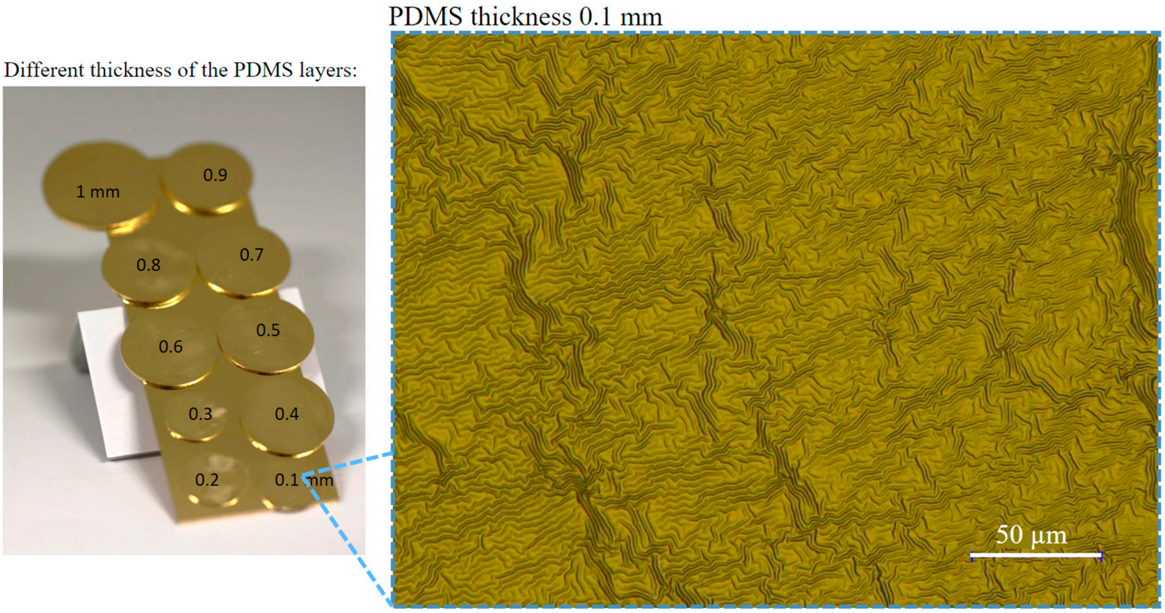

Figure S8. – Photograph of the sputtered Cr/Au on 10:1 PDMS test layers with a thickness range from 1 mm to 0.1 mm. The magnified surface view of the 0.1 mm thick PDMS with metal is on the right side. The wrinkle behavior is identical on all samples sputtered without shadow mask.

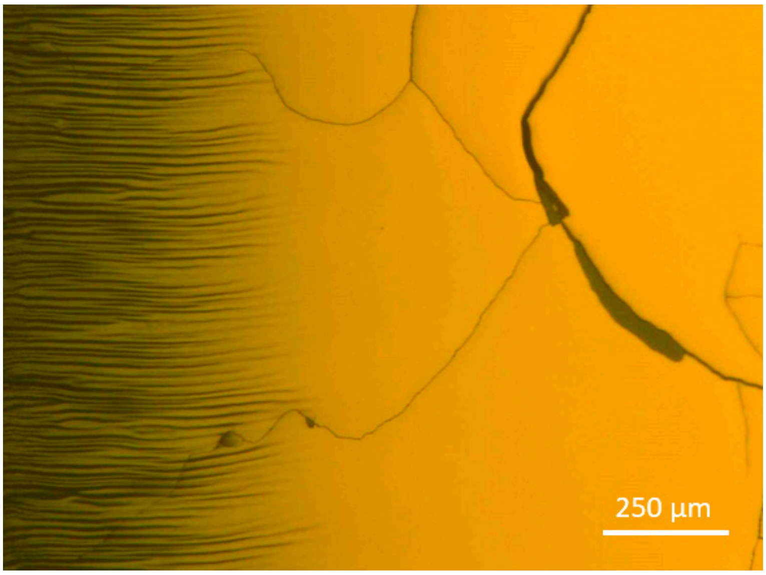

Figure S9. – Optical microscope image of the Cr/Au meander surface. Substrate is 20 μm thick free-standing 10:4 PDMS membrane. Hard PDMS has clearly seen planar and wrinkled areas. The μ-size cracks "self-heal" on the wrinkled region.

Table S1. – Detailed parameters of the Thermo-electric Meander-shaped elements on a thin freestanding PDMS membranes

**Sample**	**Meander length, mm**	**Mixing ratio (PDMS base:curing agent)**	**PDMS film thickness*, μm**	**Resistance calculated, Ohm**	**Resistance measured, Ohm**	**Wrinkles wavelength, μm**
1	29	10:1	20.4	3.46	10.8	5.1
2	48	10:1	20.37	5.73	13.5	5.07
3	29	10:1	40.09	3.46	11	5.05
4	48	10:1	40.12	5.73	14	5.08
7	29	10:4	20.1	3.46	15.4	5.33
8	48	10:4	20.2	5.73	20.5	5.34
9	29	10:4	40.51	3.46	15.42	5.34
10	48	10:4	40.4	5.73	20.7	5.36

* PDMS layer thickness was measured at three places per sample, and the average value is presented in the table

Table S2. – PDMS thickness distribution within the membrane area, where P1 is a measurement in the center, P3 near the edge and P2 in between P1 and P3.

Sample	P1, μm	P2, μm	P3, μm	Average, μm
20 μm PDMS 10:1 with short meander	20,3	20,4	20,5	20,4
20 μm PDMS 10:1 with long meander	20,34	20,37	20,4	20,37
40 μm PDMS 10:1 with short meander	40,08	40,09	40,11	40,09
40 μm PDMS 10:1 with long meander	40,11	40,12	40,14	40,12
20 μm PDMS 10:4 with short meander	40,38	40,45	40,7	40,51
20 μm PDMS 10:4 with long meander	40,29	40,31	40,6	40,4
40 μm PDMS 10:4 with short meander	19,9	20,1	20,3	20,1
40 μm PDMS 10:4 with long meander	20	20,2	20,4	20,2

Table S3. – Resistance and wavelengths of the wrinkles, for the 40 μm thick PDMS membrane with 29 mm meander, after multiple on/off cycles. The intermediate time is 5 minutes.

I, A	R_{10}, Ω	λ_{10}, μm	R_{20}, Ω	λ_{20}, μm	R_{30}, Ω	λ_{30}, μm	R_{40}, Ω	λ_{40}, μm	R_{50}, Ω	λ_{50}, μm	R_{60}, Ω	λ_{60}, μm
0	11	5.05	11	5.05	11	5.05	11	5.05	11	5.05	11	5.05
0.18	12.76	5.26	12.7	5.39	12.76	5.31	12.66	5.3	12.73	5.24	12.73	5.39
